# Relationship between maternal environment and DNA methylation patterns of estrogen receptor alpha in wild Eastern Bluebird (*Sialia sialis*) nestlings: a pilot study

**DOI:** 10.1002/ece3.2162

**Published:** 2016-06-16

**Authors:** Alexandra B. Bentz, Aubrey E. Sirman, Haruka Wada, Kristen J. Navara, Wendy R. Hood

**Affiliations:** ^1^ Poultry Science Department University of Georgia 203 Poultry Science Bldg. Athens Georigia 30602; ^2^ Department of Biological Sciences Auburn University 101 Life Science Building Auburn Alabama 36849

**Keywords:** Breeding density, diencephalon, growth rate, maternal effect, yolk testosterone

## Abstract

There is mounting evidence that, across taxa, females breeding in competitive environments tend to allocate more testosterone to their offspring prenatally and these offspring typically have more aggressive and faster‐growing phenotypes. To date, no study has determined the mechanisms mediating this maternal effect's influence on offspring phenotype. However, levels of estrogen receptor alpha (ER *α*) gene expression are linked to differences in early growth and aggression; thus, maternal hormones may alter gene regulation, perhaps via DNA methylation, of ER *α* in offspring during prenatal development. We performed a pilot study to examine natural variation in testosterone allocation to offspring through egg yolks in wild Eastern Bluebirds (*Sialia sialis*) in varying breeding densities and percent DNA methylation of CG dinucleotides in the ER *α* promoter in offspring brain regions associated with growth and behavior. We hypothesized that breeding density would be positively correlated with yolk testosterone, and prenatal exposure to maternal‐derived yolk testosterone would be associated with greater offspring growth and decreased ER *α* promoter methylation. Yolk testosterone concentration was positively correlated with breeding density, nestling growth rate, and percent DNA methylation of one out of five investigated CpG sites (site 3) in the diencephalon ER *α* promoter, but none in the telencephalon (n = 10). Percent DNA methylation of diencephalon CpG site 3 was positively correlated with growth rate. These data suggest a possible role for epigenetics in mediating the effects of the maternal environment on offspring phenotype. Experimentally examining this mechanism with a larger sample size in future studies may help elucidate a prominent way in which animals respond to their environment. Further, by determining the mechanisms that mediate maternal effects, we can begin to understand the potential for the heritability of these mechanisms and the impact that maternal effects are capable of producing at an evolutionary scale.

## Introduction

The social environment experienced by females can have formative impacts not only on her survival and reproductive success, but also on that of her offspring. One way in which this occurs is through hormone‐mediated maternal effects (i.e., embryonic exposure to environmentally elicited maternal hormones that modify offspring phenotype; Groothuis et al. 2005). Several studies have shown that females breeding in high density and/or increased social interactions allocate more testosterone to their young prenatally (Whittingham and Schwabl 2001; Mazuc et al. 2003; Pilz and Smith 2004; Dloniak et al. 2006; Hargitai et al. 2009; Bentz et al. 2013; but see von Engelhardt and Groothuis 2011); these offspring then display more‐competitive traits like faster postnatal growth during the early phase of rapid mass gain (Schwabl 1996; Eising et al. 2001; Pilz et al. 2004; Navara et al. 2005, 2006a; Cucco et al. 2008; Bentz et al. 2013; but see Gorman and Williams 2005) and increased aggressive, competitive behaviors (Strasser and Schwabl 2004; Dloniak et al. 2006; Eising et al. 2006; Partecke and Schwabl 2008; Müller et al. 2009). Maternal effects are potentially a way by which offspring can match their phenotype to current environmental conditions and the adaptive significance has been of considerable interest to evolutionary biologists (Mousseau and Fox 1998; Mcadam et al. 2002; Räsänen and Kruuk 2007). However, the link between maternal hormones and offspring phenotype is not well understood. Without knowing the mechanisms that mediate maternal effects, we cannot fully understand the heritability of this phenomenon and how it fits into a larger evolutionary framework.

Few studies have attempted to test mechanisms by which yolk testosterone influences offspring phenotype. One hypothesis is that yolk testosterone enhances growth by increasing begging rates (Schwabl 1996), but there is conflicting support (Pilz et al. 2004; Müller et al. 2012). Pfannkuche et al. (2011) found that offspring exposed to higher yolk testosterone concentrations had lower circulating testosterone and androgen receptor (AR) mRNA expression in whole brain tissue, suggesting that yolk testosterone may influence offspring phenotype by mediating changes in levels of ARs. However, testosterone does not restrictively act through ARs, but can also be converted to estrogen via aromatase and bind to the estrogen receptor (ER; Groothuis and Schwabl 2008). For example, Hegyi and Schwabl (2010) showed that when dihydrotestosterone (i.e., an unaromatizable metabolite of testosterone that only utilizes the AR) was injected into Japanese quail (*Coturnix japonica*) eggs, offspring growth was not affected. In Müller et al. (2005), egg injections of an AR antagonist also did not decrease growth in male Black‐headed Gulls (*Larus ridibundus*), a species whose growth was positively affected by yolk testosterone in a previous study (Eising et al. 2001). Thus, testosterone may act through estrogen and its ER, which are known to influence growth hormone (GH), a regulator of early growth (Meinhardt and Ho 2006; Addison and Rissman 2012). The hypothalamus (a large component of the diencephalon) expresses growth hormone‐releasing hormone (GHRH), a regulator of GH (Harvey 1985) that has estrogen‐responsive elements in its promoter (Petersenn et al. 1998), and both GH and GHRH are co‐expressed with ER*α* in the hypothalamus (Kamegai et al. 2001; Addison and Rissman 2012). Furthermore, estrogens are implicated in other phenotypic changes associated with yolk testosterone, such as aggressive behaviors (Soma 2006). Aggressive phenotype is correlated with ER*α* mRNA expression in the diencephalon and telencephalon (Filby et al. 2010; Rosvall et al. 2012), and both aromatase inhibitors and ER*α* antagonists decrease aggressive behaviors (Walters and Harding 1988; Schlinger and Callard 1990). Songbirds express ER*α* mRNA in the diencephalon and posterior telencephalon during late embryonic development irrespective of sex (Perlman and Arnold 2003). Thus, yolk testosterone may alter ER*α* expression in the diencephalon and telencephalon to cause phenotypic changes.

Epigenetic modifications have become promising candidates for explaining how environmental stimuli can influence early development. Epigenetic effects create stable changes in gene expression without altering DNA sequence; for example, adding methyl groups to cytosines at CG dinucleotides (i.e., CpG sites) in gene promoters typically suppresses gene expression (Holliday 1994). Organisms are most susceptible to epigenetic modifications in early development (Vickaryous and Whitelaw 2005). Most notably, sexual differentiation of the brain is thought to be influenced by endogenous prenatal steroid‐induced alteration of steroid‐receptor gene, primarily ER*α*, methylation (Schwarz et al. 2010), and histone acetylation (Matsuda et al. 2011). Moreover, it is during early development that animals are most dependent on maternal factors and it stands to reason that maternal effects could influence epigenetic patterns. Maternal influence in the early postnatal environment can affect DNA methylation status in offspring (Weaver et al. 2004; Murgatroyd et al. 2009), specifically, DNA methylation of the ER*α* (Champagne et al. 2006). Studies concerning prenatal epigenetic maternal effects often focus on the maladaptive effects of maternal exposure to pollutants, poor nutrition, or stress (Feil and Fraga 2012). However, animals are exposed to exogenous testosterone during prenatal development (Parsons 1970; von Engelhardt et al. 2009), yet few studies examine this effect. One study (Mori et al. 2010) did show a relationship between prenatal exposure to naturally occurring exogenous inputs of testosterone and decreased ER*α* DNA methylation and greater ER*α* mRNA expression in the hypothalamus along with increased aggression. This study supports the hypothesis that maternal social environment could affect offspring phenotype through prenatal testosterone‐induced epigenetic modifications.

**Figure 1:**
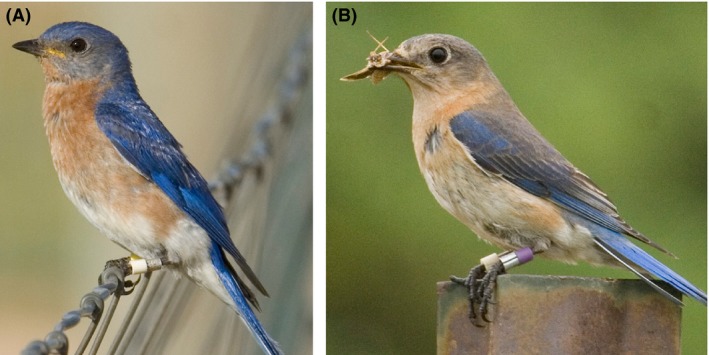
Picture of (A) male and (B) female Eastern Bluebirds, *Sialia sialis* (photo courtesy of Geoffrey E. Hill).

In the present pilot study, we investigated relationships between natural variation in the competitive environment experienced by Eastern Bluebirds (*Sialia sialis*; Fig. 1) and their yolk testosterone allocation, offspring growth, and ER*α* DNA methylation in offspring brain tissue to help inform and promote future experimental studies. Eastern Bluebirds are obligate secondary cavity nesters and are limited by available cavities, causing intense competition for cavities in high breeding densities (Pinkowski 1976; Parren 1991; Gowaty and Plissner 1998). Because yolk testosterone concentrations have been linked to competitive environment and influence offspring growth rates and aggression in other species (von Engelhardt and Groothuis 2011), we hypothesized that (1) higher breeding densities would be positively correlated with testosterone allocation to egg yolks and (2) that exposure to high yolk testosterone concentrations would be related to faster growth rates in Eastern Bluebirds. Further, because previous work cited above indicates that testosterone may affect offspring phenotype via aromatization to estrogen, we further hypothesized that (3) higher yolk testosterone concentrations would be associated with decreased ER*α* DNA methylation in the diencephalon and posterior telencephalon in offspring and (4) patterns of ER*α* DNA methylation would negatively correlate with offspring growth rates.

## Materials and Methods

### Study population

We monitored 142 nest boxes placed throughout Auburn, AL (32.5978°N, 85.4808°W) in 2011 from March through May. We were able to observe 26 Eastern Bluebird pairs and we collected the fourth egg from the first breeding attempt (*n* = 19; seven eggs were either unable to be collected prior to incubation or lost in processing). The fourth egg is representative of the entire clutch because bluebirds display low within‐clutch variation in yolk testosterone (Navara et al. 2006b; Duckworth et al. 2015). We then measured body mass (±0.01 g) of nestlings from nests that successfully hatched (*n* = 25; one nest was lost to predation) on days two, five, eight, and 11 posthatch, and fledging mass at 14 days posthatch. Growth followed a sigmoidal pattern and reached a plateau around day 11. Therefore, the growth rate for each nestling was derived from the slope of a linear regression of nestling mass on days 2–11 posthatch to ensure we captured the period of rapid, linear growth (for all nestlings: *r* ^2^ > 0.80). We sacrificed one randomly selected 14‐day‐old nestling from 17 different nests (six male and 11 female nestlings) for DNA methylation analyses. Brains were immediately dissected and postfixed in buffered 4% paraformaldehyde for 8 days at 4°C. Brains were then cryoprotected in 30% sucrose solution until they were fully penetrated by the cryoprotectant (~48 h) before they were frozen on ground dry ice and stored at −80°C. To dissect the diencephalon and posterior telencephalon, we discarded the cerebellum and performed a punch biopsy of the underlying diencephalon, acquiring primarily hypothalamic tissue, and collected the portion of the posterior telencephalon consisting of the nucleus taeniae of the amygdala according to the revised songbird brain atlas by Reiner et al. (2004). All procedures were conducted according to protocol #2011‐1887 approved by the Auburn University Institutional Animal Care and Use Committee.

Breeding density was measured using Google Earth Pro to map the GPS location of all nest boxes at the field site and to create polygons encompassing each nest box territory. Polygon territories were defined as useable habitat (i.e., open meadows that have less than 50% tree cover) within a 300 m radius around each nest box as used in Duckworth et al. (2015). The area of each polygon was measured in hectares and the number of occupied nest boxes within each territory was counted to create a density measure of occupied nest boxes per hectare. We considered nest boxes within the territory occupied if a pair was present during the 6 days prior to the focal pair laying their fourth egg because yolk deposition can occur 6 days prior to an egg being laid (Navara et al. 2006b).

### Yolk hormone analysis

Yolk testosterone was extracted from homogenized yolk samples with a double ether extraction followed by liquid column chromatography according to methods described by Schwabl (1993). Briefly, 50 mg of yolk was weighed and vortexed with 1000 *μ*L of deionized water. Next, 3 mL of petroleum:diethyl ether (30:70 vol/vol) was added, the mixture was vortexed for 30 sec and was allowed to settle for 20 min. Samples were then snap frozen and the supernatant was poured off and dried. The sample was reconstituted in 1 mL 10% ethyl acetate in isooctane and steroids were separated using celite column chromatography. Testosterone was eluted in 20% ethyl acetate in isooctane. Testosterone was quantified with a standard competitive‐binding radioimmunoassay using anti‐testosterone (MP Biomedicals, Solon, OH) as described in Wingfield and Farner (1975). Anti‐testosterone had a crossreactivity of 100% with testosterone, 18.75% with dihydrotestosterone, 3% with androstanediol, and <1% with all other steroids. Average recoveries were 89% and average intraassay variation was 8%.

### Promoter identification

The Eastern Bluebird genome has not yet been sequenced and annotated. However, regions of gene regulation such as promoters are generally highly conserved across species (Carninci et al. 2006), so to identify a putative promoter region for the ER*α* gene in Eastern Bluebirds we used the most closely related songbird genome that has been fully sequenced as a starting point for comparison across species (i.e., Zebra Finch, *Taeniopygia guttata*; Warren et al. 2010). We identified 4000 bp of 5′ flanking region and the 434 bp exon one of the Zebra Finch ER*α* gene using Ensembl (ENSTGUG00000011249) and compared sequence homology using NCBI BLAST (http://www.ncbi.nlm.nih.gov/BLAST/). We only compared sequences with at least 96% similarity that were also from the order Passeriformes and predicted to be part of the ER*α* gene; this included nine species: *Geospiza fortis*,* Corvus cornix, Corvus brachyrhynchos*,* Acanthisitta chloris*,* Serinus canaria*,* Manacus vitellinus*,* Ficedula albicollis*,* Zonotrichia albicollis*, and *Pseudopodoces humilis*. We then designed primers for the most highly conserved region. The forward primer (5′‐ACCCAGACACACAAACATAC‐3′) and reverse primer (5′‐GCAGTGAGCAAGGAACAT‐3′) were designed with PrimerQuest (Integrated DNA Technologies, Inc., Coralville, IA).

DNA was extracted from homogenized posterior telencephalon brain tissue using a DNeasy Blood and Tissue Kit (Qiagen, Germantown, MD) according to the manufacturer's protocol. PCR was performed in 50 *μ*L which contained 1 *μ*L of DNA template, 1 *μ*L each of the forward and reverse primers, and 25 *μ*L of PCR Master Mix, 2x (Promega, Madison, WI). The PCR conditions were an initial denaturation of 94°C for 2 min then 35 cycles of 94°C for 30 sec, 55°C for 30 sec, and 72°C for 30 sec with a final extension step of 72°C for 10 min. PCR products were visualized under ultraviolet light after 2% agarose gel electrophoresis. PCR products were purified using DNA Clean and Concentrator (Zymo Research, Irvine, CA) and sequenced in both directions at the Auburn University Genomics and Sequencing Lab. Nucleotide sequences were read and assembled using the software Chromas Lite (Technelysium Pty. Ltd., South Brisbane, Australia). We identified potential transcription factor binding sites (TFBSs) on the putative Eastern Bluebird ER*α* promoter using MatInspector (Genomatix; Cartharius et al. 2005). We checked for corresponding TFBSs in the annotated Zebra Finch promoter. We included binding sites of all TFBS that have been identified in brain tissue according to MatInspector (for a list of all potential TFBSs see Table S1).

### DNA methylation analysis

We performed bisulfite‐PCR on a 339 bp region of the 5′ flanking region immediately upstream of exon one (i.e., putative promoter region) in both the diencephalon and posterior telencephalon. DNA extraction and bisulfite conversion were performed simultaneously with 0.1 mg of homogenized tissue using the EZ DNA Methylation Kit (Zymo Research) according to the manufacturer's protocol for fixed tissue. The forward primer (5′‐GAAAAATTAAAAGATTAGTAAGAATGAAGT‐3′) and reverse primer (5′‐AAACAAAAAACATATCTACTTTCACT‐3′) for bisulfite‐converted DNA were designed using Methyl Primer Express (Applied Biosystems, Foster City, CA, USA). PCR was performed in 25 *μ*L which contained 4 *μ*L of bisulfite‐converted DNA template, 1 *μ*L each of the forward and reverse primers, and 12.5 *μ*L of Zymo*Taq* DNA Polymerase (Zymo Research). The PCR conditions were an initial denaturation of 95°C for 10 min then 35 cycles of 95°C for 30 sec, 52°C for 30 sec, and 72°C for 60 sec with a final extension step of 72°C for 7 min. PCR products were visualized under ultraviolet light after 2% agarose gel electrophoresis. Due to low DNA concentrations, we used 1 *μ*L of PCR product as the template for a second round of PCR using the same primer pair and only 30 cycles. PCR products were visualized, purified, sequenced, and assembled as previously described. To determine the efficiency of the bisulfite conversion, we calculated conversion rate for each sample as the percent of cytosines not at a CpG site that were converted to thymine (Jiang et al. 2010). Percent DNA methylation for each CpG site was calculated as the peak height of cytosine divided by the sum of the peak height for cytosine and thymine (Jiang et al. 2010). Calculating percent DNA methylation using direct bisulfite‐PCR sequencing has been shown to produce comparable results to that of pyrosequencing and bisulfite‐cloning sequencing (Jiang et al. 2010).

### Statistical analyses

For hypotheses one and two, we first performed a linear regression to determine if variation in yolk testosterone concentration could be explained by breeding density. Next, we used a linear mixed effects model to determine if either growth rate or fledging mass at day 14 was correlated with yolk testosterone concentration while controlling for offspring sex and brood size. For the mixed effects model, nest box ID was the random effect to account for the fact that some nestlings came from the same brood, and this model was fit with restricted maximum likelihood using the *nlme* package in R (Pinheiro et al. 2015).

To address hypotheses three and four, we performed separate linear regressions for each CpG site in the diencephalon and telencephalon to test (1) if yolk testosterone concentration was correlated with percent DNA methylation of each CpG site (*n* = 10; of the 17 brain samples, only 10 were from nests in which a yolk sample had been collected) and (2) if percent DNA methylation of CpG sites was related to growth phenotype while accounting for brood size and offspring sex (*n* = 17 for fledging mass analysis and *n* = 16 for growth rate analysis; one nest was only visited twice and growth rate could not be confidently calculated). We chose not to use *P*‐value adjustments to correct for multiple testing when examining percent DNA methylation of the five CpG sites within each brain region because there were only five comparisons and our small sample size already makes us conservative with type I error fixed at *α *= 0.05 and particularly susceptible to type II error, which *P*‐value corrections would exacerbate (Rothman 1990; Johnson 1999). Additionally, we tested all model residuals for outliers using the Grubbs' outlier test (Grubbs 1950) with the *outliers* package in R (Komsta 2011) because small sample sizes, such as ours, are sensitive to observations that have high leverage or are too influential making ordinary least squares regression methods inappropriate. When outliers were detected, we used a robust linear regression to calculate estimates that are not as strongly affected by outliers (Rousseeuw and Leroy 1987). Specifically, we employed an SMDM‐type regression estimator that performs well with small sample sizes (Koller and Stahel 2011) using the *robustbase* package in R (Rousseeuw et al. 2015). We also performed correlations with percent DNA methylation between and within CpG sites in the diencephalon and telencephalon to look for inter‐ and intrarelationships. All statistical analyses were performed with R version 3.0.1 (R Development Core Team 2013). All means are followed by the standard error.

## Results

### Breeding density, yolk testosteorne, and offspring growth

**Figure 2:**
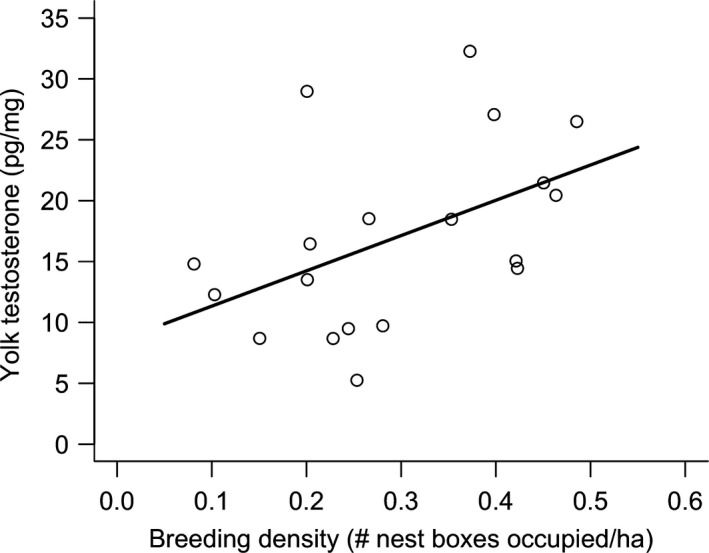
Correlation between Eastern Bluebird breeding densities (i.e., the number of occupied nest boxes per area of useable habitat within a 300 m radius of each box) and yolk testosterone concentrations in the fourth egg.

On average, there were 4.7 (±0.4; 1–11) nest boxes in each territory, of which 78.8% (±3.6; 40–100%) were occupied. The number of occupied nest boxes per hectare was a significant and positive predictor of yolk testosterone (*β *= 28.98 ± 12.99, *r* ^2^ = 0.23, *F*_1,17_ = 4.98, *P *=* *0.04; Fig. 2). Growth rate was significantly and positively correlated with yolk testosterone (*β *= 0.01 ± 0.01, *F*_1,14_ = 7.55, *P *=* *0.02) and negatively correlated with brood size (*β *= −0.11 ± 0.04, *F*_1,14_ = 6.20, *P *=* *0.03), but nestling sex was not a significant predictor of nestling growth rate (*P *=* *0.41). Neither yolk testosterone (*P *=* *0.52), sex (*P *=* *0.16), nor brood size (*P *=* *0.59) was significantly correlated with fledging mass at day 14.

### Promoter identification

We compared the 5′ flanking region and exon one of the ER*α* gene across 10 avian species and found that the most highly conserved region of the 4434 bp sequence was exon one and 216 bp of the immediately upstream 5′ flanking region. This highly conserved region also coincided with the promoter region of the Zebra Finch ER*α* gene identified by ElDorado, the Genomatix genome annotation (Cartharius et al. 2005). Using primers designed to detect this region, we obtained a 656 bp fragment from Eastern Bluebird telencephalon tissue which we aligned with the Zebra Finch genome using the University of Santa Cruz (USCS) Genome Browser BLAT program (http://genome.ucsc.edu/) and found that it had a 99.6% identity with the promoter of the Zebra Finch ER*α* gene. Using the 656 bp region as a template, we aimed to determine the DNA methylation pattern of the most highly conserved region and likely promoter (i.e., 216 bp upstream of exon one) of the Eastern Bluebird ER*α* gene.

### Yolk testosterone and DNA methylation

**Figure 3:**
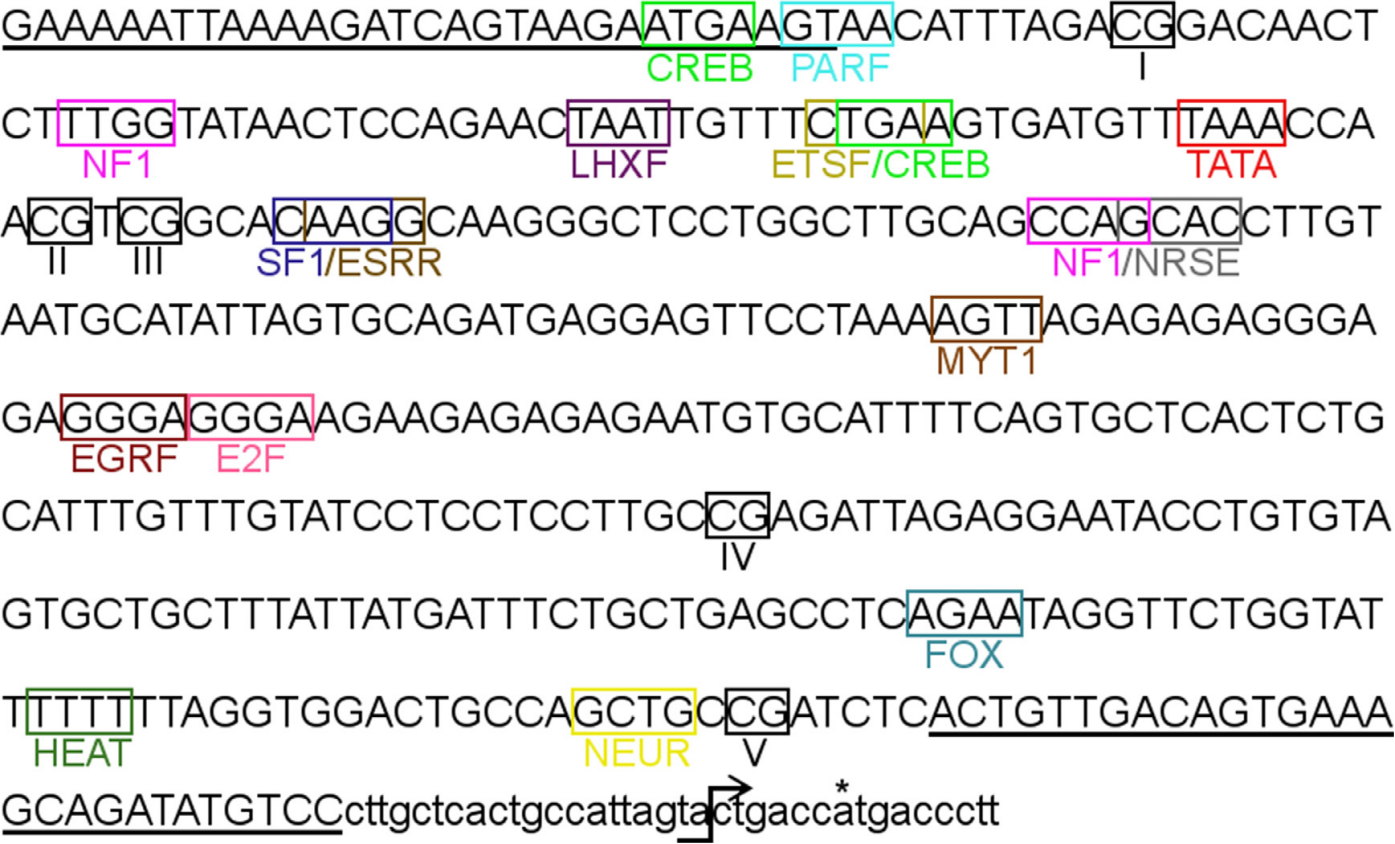
Sequence for the putative promoter region of estrogen receptor alpha in the Eastern Bluebird. The core conserved sequences (four nucleotides) for transcription factor binding sites and CpG sites (labeled with roman numerals I–V) are indicated in colored and black boxes, respectively. Upper case letters were sequenced from Eastern Bluebird brain tissue and lower case letters are from Zebra Finches (ENSTGUG00000011249), so that the potential transcription start site could be shown. The transcription start site is indicated with an arrow. A potential translation initiation codon, ATG, is indicated with an asterisk. The sequences of the primer pairs used for bisulfite‐PCR are underlined. CREB: cAMP‐response element binding protein. ESRR: estrogen‐related receptor alpha. E2F: E2F transcription factor 7. MYT1: myelin transcription factor 1. EGRF: early growth response factor. NRSE: neuron‐restrictive silencer factor. ETSF: ETS1 factor. LHXF: LIM homeodomain transcription factor. PARF: PAR‐domain basic leucine zipper transcription factor. NF1: nuclear factor 1. TATA: TATA box. SF1: vertebrate steroidogenic factor 1. NEUR: neuroD. FOX: forkhead domain factor. HEAT, heat shock factor.

**Figure 4:**
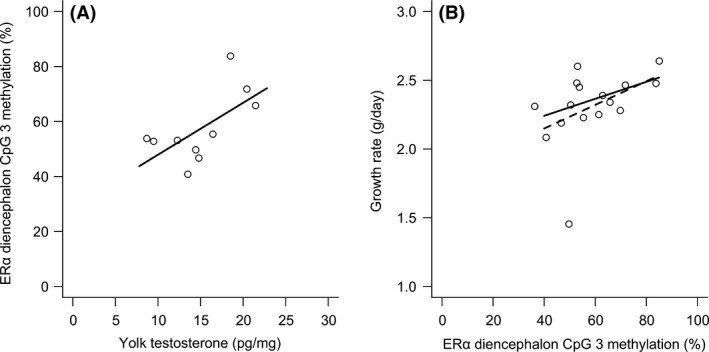
Relationships between (A) yolk testosterone concentration and percent DNA methylation of CpG site 3 in the putative promoter region of estrogen receptor alpha (ER *α*) in the diencephalon (solid line is linear regression line) and (B) percent DNA methylation of ER *α* CpG site 3 in the diencephalon and nestling growth rate (solid line is robust regression line and dashed line is linear regression line).

Bisulfite‐PCR produced a 339 bp fragment in the putative promoter region of the Eastern Bluebird ER*α* gene, in which five CpG sites and all potential TFBSs specific to brain tissue were identified (Fig. 3; for a list of all potential TFBSs see Table S1). Our bisulfite conversion rate was 91.48% (± 0.87) in the diencephalon and 93.20% (±0.83) in the telencephalon. Average percent DNA methylation of CpG sites within the diencephalon was as follows: CpG site 1 = 68.91% (±4.36), CpG site 2 = 56.03% (±2.98), CpG site 3 = 58.38% (±3.29), CpG site 4 = 96.44% (±1.42), and CpG site 5 = 81.46% (±1.75). Average percent DNA methylation of CpG sites within the telencephalon was as follows: CpG site 1 = 65.93% (±4.16), CpG site 2 = 51.62% (±3.92), CpG site 3 = 49.59% (±3.73), CpG site 4 = 97.73% (±0.82), and CpG site 5 = 74.57% (±3.97). Contrary to what we hypothesized, yolk testosterone concentration was significantly and positively correlated with percent DNA methylation of CpG site 3 in the ER*α* in the diencephalon (Fig. 4A), but not with any other CpG sites (Table 1). Percent DNA methylation of ER*α* was not strongly correlated between CpG sites either within or between brain regions in an individual (all *r *<* *0.63).

**Table 1:** Linear regression analyses of the percent DNA methyaltion of each CpG site in the putative promoter region of estrogen receptor alpha in 14‐day‐old Eastern Bluebird offspring in the diencephalon and telencephalon with yolk testosteorne concentration as the predictor variable. Only one significant outlier was detected using Grubbs' outlier test in the residuals of the regression between percent DNA methylation of telencephalon CpG site 4 and yolk testosterone (*G* = 2.41, *P* < 0.01); however, whether linear regression (*P* = 0.72) or robust regression (*P *=* *0.91) was used the significance of the outcome did not change, so the linear regression results are presented in the table

Brain Region	CpG	*β* (SE)	df	*F*	*P*
Diencephalon	1	0.16 (1.40)	1, 8	0.01	0.91
	2	0.01 (0.46)	1, 8	<0.01	0.99
	3	1.89 (0.82)	1, 8	5.34	**0.049**
	4	−0.20 (0.38)	1, 8	0.28	0.61
	5	−0.11 (0.74)	1, 8	0.02	0.88
Telencephalon	1	−0.65 (1.47)	1, 8	0.20	0.67
	2	−0.74 (1.60)	1, 8	0.21	0.66
	3	−1.71 (1.11)	1, 8	2.37	0.16
	4	0.13 (0.34)	1, 8	0.14	0.72
	5	−0.14 (1.54)	1, 8	0.01	0.93

Bold values represent *P *< 0.05.

### Nestling growth and DNA methylation

We tested if percent methylation of CpG site 3 in the diencephalon (the only CpG site that was correlated with yolk testosterone concentration) could explain variation in growth rate. Grubbs' outlier test indicated that an outlier was present in the residuals (*G* = 3.03, *P *<* *0.01), thus, we used robust linear regression methods and found that growth rate was significantly and positively correlated with percent methylation of CpG site 3 in the diencephalon (robust regression: *β *= 0.01 ± 0.002, *t*_3,12_ = 2.71, *P *=* *0.02; linear regression: *β *= 0.01 ± 0.004, *t*_3,12_ = 1.40, *P *=* *0.19; Fig. 4B). Growth rate was also significantly, negatively correlated with brood size (robust regression: *β *= −0.12 ± 0.04, *t*_3,12_ = −3.11, *P *=* *0.01; linear regression: *β *= −0.19 ± 0.07, *t*_3,12_ = −2.57, *P *=* *0.02), but not offspring sex (robust regression: *P *=* *0.32; linear regression: *P *=* *0.63). Fledging mass at day 14 was not correlated with CpG site 3 in the diencephalon; there was an outlier (G = 2.49; *P *=* *0.05), but this did not change the significance of the outcome (robust regression: *P *=* *0.94; linear regression: 0.90).

## Discussion

Our findings agree with previous studies showing that yolk testosterone is positively correlated with breeding density and offspring postnatal growth (see Introduction). Our preliminary data also suggest that ER*α* DNA methylation in the diencephalon may play a role in linking maternal environment and growth rate, although our data should be interpreted cautiously given our limited sample size. We found a positive correlative relationship between yolk testosterone concentration and DNA methylation of CpG site 3 in the ER*α* promoter in the diencephalon. Furthermore, ER*α* CpG site 3 percent DNA methylation in the diencephalon was positively related to growth rate. Our analysis of the putative promoter region indicated that CpG site 3 was in close proximity to several potential TFBS, one of which was a TATA box (i.e., a strong initiator of transcription; Carninci et al. 2006; Kitazawa and Kitazawa 2007), signifying that it could have important implications for transcriptional control. While we did not measure ER*α* mRNA expression, Fürst et al. (2012) showed that it is possible for its expression to be decreased by greater methylation of a single CpG site. Regardless, future experimental manipulations should be performed to investigate the directionality and causality of the patterns found in our correlative study.

There are several potential explanations for why we found correlative relationships between percent DNA methylation at CpG 3 in the diencephalon, yolk testosterone, and growth rate. One of the more intriguing explanations to explore in future research is the relationship between ER*α*, GH, and growth. One of the main factors known to regulate food intake and growth in birds is GH (Buntin and Figge 1988). We had predicted decreased methylation of ER*α*, but estrogen can have both positive (Hassan et al. 2001; Yan et al. 2004) and negative effects (Lam et al. 1996; Petersenn et al. 1998) on GH, likely due to variation in estrogen concentration and GH's ability to autoregulate (Tennenbaum 1980; Bagamasbad and Denver 2011). For example, Childs et al. (2005) showed that low concentrations of estrogen stimulate more GH cells to display GHRH‐binding sites. In one of the few avian studies, Hall et al. (1984) incubated chicken pituitary glands in hypothalamic extract and measured GH secretion with and without estrogen priming and found that estrogen‐primed pituitaries were less sensitive to GH‐releasing activity. Furthermore, the effects of estrogen on GH are blocked if an ER*α* antagonist is administered (Avtanski et al. 2014), providing evidence that estrogen regulates GH via ER*α*. Therefore, lower levels of estrogen and ER*α* may increase cellular sensitivity to hypothalamic GHRH during postnatal growth and if ER*α* expression was indeed lowered in our study then this could help explain the positive correlation with growth rate.

Additionally, concentrations of GH in birds peak during the period of rapid, early growth and then decline, remaining low throughout adulthood (Scanes and Balthazart 1981; Scanes et al. 1992; Schew et al. 1996). This may explain why we found a relationship between percent DNA methylation and early growth rate, but not fledging mass, which was measured approximately 3 days after peak mass was reached. Thus, the relationship we found between ER*α* DNA methylation in the diencephalon and growth could be related to the interaction between ER*α* and GHRH in this brain region. However, our study was purely correlative and further experimentation is needed to fully test this idea.

The patterns we found between yolk testosterone, growth, and percent ER*α* DNA methylation in the diencephalon were not found in the telencephalon, and it is not clear why only the diencephalon would show these relationships. It may simply be that we lacked the power to detect these relationships and future studies should not be deterred from investigating this brain region in the context of yolk testosterone. However, another possibility is that, in birds, the diencephalon has greater levels of aromatase activity than the telencephalon (Balthazart et al. 1990) and testosterone can suppress aromatase activity (Bagamasbad and Denver 2011). Exposure to high testosterone concentrations early in development may lead to a permanent downregulation of estrogenic activity in the diencephalon, but not the telencephalon. Furthermore, the telencephalon is implicated in social behaviors but not growth (Goodson 2005), supporting a lack of a relationship between ER*α* DNA methylation in the telencephalon and growth.

The other well‐studied effect of yolk testosterone on offspring phenotype is an increase in aggressive, competitive behaviors (see Introduction). We were unable to measure aggression due to the fact that Eastern Bluebirds are altricial and do not express explicit aggressive behaviors until after fledging. However, our findings may still be tentatively applied to the effects of yolk testosterone on aggression. Rosvall et al. (2012) showed that more aggressive Dark‐eyed Juncos (*Junco hyemalis*) had greater expression of ER*α* in the telencephalon but lower expression in the hypothalamus. This mirrors our findings, in that we found greater DNA methylation in the diencephalon, which is suggestive of lower expression in individuals exposed to more yolk testosterone (Fürst et al. 2012).

An alternative explanation for our findings could be that they are an artifact of our small sample size or other unmeasured factors. We chose not to use *P*‐value adjustments when investigating relationships between percent methylation at the five CpG sites and yolk testosterone in each brain region because of the trade‐off between type I and type II error it would require. Small sample sizes, such as ours, are particularly prone to type II errors (Johnson 1999), and adjusting *P*‐values would decrease the possibility of type I errors at the expense of type II errors (Rothman 1990). In a pilot study meant to prompt future research, we felt that the cost of a false negative was much greater than that of a false positive. Therefore, the significant relationships we found with percent methylation at CpG site 3 in the diencephalon could be a result of type I error; however, it is more likely that there are more biologically significant relationships than we were able to statistically detect. There are also other components of yolk that are affected by breeding density and/or social interactions that could affect growth that we did not measure in this study. For example, lesser black‐backed gulls (*Larus fuscus*) increase yolk carotenoids with frequency of social interactions (Verboven et al. 2005) and black‐headed gulls (*Larus ridibundus*) increase yolk antibodies with breeding density (Müller et al. 2004); however, these relationships have yet to be found in passerines (Hargitai et al. 2009; Safran et al. 2010; Remeš et al. 2011). Birds nesting in higher densities also tend to deposit less corticosterone in their egg yolks (Love et al. 2008; but see Bentz et al. 2013). Nevertheless, yolk androgens are the best‐studied effect of breeding density and/or social interactions (von Engelhardt and Groothuis 2011) and, despite the correlative nature of our study, the best candidate for the effects we measured.

## Conclusions

Hormone‐mediated maternal effects potentially cause adaptive changes in offspring phenotype and, while past studies have examined what causes females to vary prenatal hormones and what offspring phenotypes arise as a consequence, it is still unclear how maternal hormones exert their influence. The preliminary data presented herein shed light on the potential mechanisms that mediate the effect of environmentally elicited maternal testosterone on offspring phenotype and suggest avenues for future studies. Thus far, few studies (see Müller et al. 2005) have injected eggs with an AR antagonist and measured growth. Future studies could administer AR or ER*α* antagonists or aromatase inhibitors into yolk along with testosterone to directly test the hypothesis that yolk testosterone acts through ER*α*. Also, because we sacrificed nestlings on day 14, our correlative study is unable to separate whether percent DNA methylation was prenatally programmed and influenced growth or if postnatal growth programmed percent DNA methylation. While most epigenetic programming occurs prenatally (Vickaryous and Whitelaw 2005), postnatal experiences have been shown to influence DNA methylation patterns (Weaver et al. 2004; Champagne et al. 2006; Murgatroyd et al. 2009). Thus, while our findings do give a natural context, it is important for future studies to experimentally test these ideas while incorporating a cross‐foster design. Finally, further analysis of the structure of the ER*α* promoter in passerines is necessary to provide insights into the mechanisms that regulate the expression of ER*α* and to provide tools to investigate the relevance of these mechanisms to maternal effects. Ultimately, by determining the mechanism by which maternal effects influence offspring phenotype, we can better understand the potential for the heritability of these mechanisms and the impact that maternal effects are capable of producing at an evolutionary scale.

## Data accessibility

All data associated with this manuscript are archived in GenBank (accession number KT852372) and Dryad (doi:10.5061/dryad.4351q).

## Conflict of Interest

None declared.

## Supporting information


**Table S1.** All potential transcription factor binding sites (matrix similarity >72%) on the putative Eastern Bluebird (*Sialia sialis*) ER*α* promoter region according to MatInspector (Genomatix; Cartharius et al. 2005).
